# Smoking and Provision of Smoking Cessation Interventions among Inpatients with Acute Coronary Syndrome in China: Findings from the Improving Care for Cardiovascular Disease in China-Acute Coronary Syndrome Project

**DOI:** 10.5334/gh.784

**Published:** 2020-10-23

**Authors:** Guoliang Hu, Mengge Zhou, Jing Liu, Sidney C. Smith, Changsheng Ma, Junbo Ge, Yong Huo, Gregg C. Fonarow, Yongchen Hao, Jun Liu, Kathryn A. Taubert, Louise Morgan, Na Yang, Yuhong Zeng, Yaling Han, Dong Zhao, on behalf of the CCC-ACS Investigators

**Affiliations:** 1 Department of Epidemiology, Beijing Anzhen Hospital, Capital Medical University, Beijing Institute of Heart, Lung and Blood Vessel Diseases, Beijing, CN; 2 Division of Cardiology, University of North Carolina, Chapel Hill, NC, US; 3 Department of Cardiology, Beijing Anzhen Hospital, Capital Medical University, Beijing, CN; 4 Department of Cardiology, Shanghai Institute of Cardiovascular Diseases, Zhongshan Hospital, Fudan University, Shanghai, CN; 5 Department of Cardiology, Peking University First Hospital, Beijing, CN; 6 Division of Cardiology, Geffen School of Medicine at University of California, Los Angeles, CA, US; 7 Department of International Science, American Heart Association, Basel, CH; 8 International Quality Improvement Department, American Heart Association, Dallas, TX, US; 9 Cardiovascular Research Institute and Department of Cardiology, General Hospital of Northern Theater Command, Shenyang, Liaoning, CN

**Keywords:** acute coronary syndrome, smoke, prevalence, smoke cessation intervention, in-hospital outcome

## Abstract

**Highlights:**

- Over half of male acute coronary syndrome patients were smokers in China.
- Smoking was associated with higher risk of critical cardiac symptoms at admission.
- Only 35.3% of smoking patients received smoking cessation interventions in China.

**Background:**

Smoking cessation is recognized as an effective and cost-effective strategy for improving the prognosis of patients with coronary heart disease. Despite this, few studies have evaluated the smoking prevalence and provision of smoking cessation interventions among patients with acute coronary syndrome (ACS) in China.

**Objectives:**

To evaluate the smoking prevalence, clinical conditions and in-hospital outcomes associated with smoking, and the provision of smoking cessation interventions among ACS patients in China.

**Methods:**

This registry study was conducted using data from the Improving Care for Cardiovascular Disease in China project, a collaborative nationwide registry of the American Heart Association and the Chinese Society of Cardiology. Our study sample comprised 92,509 ACS inpatients admitted between November 2014 and December 2018. A web-based data collection platform was used to report required data.

**Results:**

Smoking prevalence among male and female ACS patients was 52.4% and 8.0%, respectively. Patients younger than 45 years had the highest smoking rate (men: 68.0%; women: 14.9%). Compared with non-smokers, smokers had an earlier onset age of ACS and a greater proportion of severe clinical manifestations at admission, including ST-elevation myocardial infarction (67.8% versus 54.8%; p < 0.001) and substantially elevated myocardial injury markers (86.1% versus 83.0%; p < 0.001). After multivariable adjustment, smoking was associated with higher risk of critical cardiac symptoms at admission (OR = 1.14, 95% CI: 1.08–1.20; p < 0.001) and had no direct association with in-hospital outcomes (OR = 0.93, 95% CI: 0.84–1.02; p = 0.107) of ACS patients. Of 37,336 smokers with ACS, only 35.3% received smoking cessation interventions before discharge. There was wide variation in provision of smoking cessation interventions across hospitals (0%–100%).

**Conclusions:**

Smoking is highly prevalent among ACS patients in China. However, smoking cessation interventions are not widely adopted in clinical practice in China as part of formal treatment strategies for ACS patients, indicating an important target for quality improvement.

**Clinical Trial Registration:**

URL: http://www.clinicaltrials.gov. Unique identifier: NCT02306616.

## Introduction

Coronary heart disease (CHD) is a leading cause of both death and premature death in China [1]. Patients with acute coronary syndrome (ACS), a severe form of CHD, are at very high risk of CHD mortality and represent an important target population for secondary prevention strategies. Of these, smoking cessation is recognized as an effective and cost-effective strategy for CHD patients with the potential to reduce an individual’s mortality risk by around 36%, compared with a 29% reduction for statins, a 23% reduction for either beta-blockers or angiotensin-converting enzyme inhibitors (ACEIs), and a 15% reduction for aspirin [2]. All guidelines on the management of ACS strongly recommend smoking cessation interventions for smokers before discharge [3,4,5,6,7,8]. The time of hospitalization should represent a ‘teachable moment’ for ACS patients who are smokers. One systematic review found that intensive smoking cessation interventions initiated during hospitalization were effective at increasing cessation rates following discharge [9]. However, few studies systematically evaluated the magnitude of smoking problems and the provision of smoking cessation interventions among ACS inpatients in China. The latest study based on the China Acute Myocardial Infarction registry only reported an overall current smoking rate (43.7%) of acute myocardial infarction (AMI) patients in 2013, without further subgroup analysis [10]. The China Patient-centered Evaluative Assessment of Cardiac Events Retrospective Study of Acute Myocardial Infarction (China PEACE-Retrospective AMI Study) showed that only 8.8% of AMI patients received smoking cessation interventions before discharge in 2011 [11]. With the implementation of strategies for smoking cessation among the general population in China in recent years, it is unclear whether the smoking rate and the provision of smoking cessation interventions have changed among ACS patients. Therefore, national studies are still needed to systematically assess the smoking rate and the provision of smoking cessation interventions before discharge among inpatients with ACS.

Smoking is a well-recognized risk factor for the development of ACS, however, the results of some previous studies on the relationship between smoking and in-hospital outcomes of ACS patients are still controversial [12,13,14,15]. The differences of results in previous studies on the relationship between smoking and in-hospital outcomes of ACS patients may result from the differences in the length of in-hospital stay (mean 3.5–8.6 days) of patients, inconsistent definition of in-hospital outcomes, and differences in confounding factors adjusted in multivariable analysis model [12,13,14,15]. It is important to clarify this issue by further observational study among ACS patients.

This study therefore aimed to evaluate the smoking prevalence among ACS patients, clinical conditions and in-hospital outcomes associated with smoking, and the proportion of smokers receiving smoking cessation interventions before discharge, using data from the Improving Care for Cardiovascular Disease in China-ACS Project (CCC-ACS Project).

## Methods

### Study design

The CCC-ACS project, launched in 2014, is a large nationwide registry and quality-improvement study with an ongoing database focusing on quality of ACS care. It is a collaborative initiative of the American Heart Association and the Chinese Society of Cardiology. Details of the design and methodology of the CCC project have been published [16]. In brief, the study included 158 tertiary hospitals and 82 secondary hospitals from 30 provinces, autonomous regions, or municipalities of different geographic-economic levels in China. Trained data abstractors at participating hospitals reported the required data from medical records via a web-based data-collection platform (Oracle Clinical Remote Data Capture; Oracle Corp, Redwood City, CA, USA). Third-party clinical research associates performed quality audits to ensure that cases were reported consecutively rather than selectively. Around 5% of reported cases were randomly selected, and their data were compared with the original medical records to ensure accuracy and completeness (Supplementary Methods).

### Study population

A total of 92,509 ACS inpatients, identified using their principal discharge diagnosis, were enrolled across China from November 2014 to December 2018. ACS was defined according to guidelines issued by the Chinese Society of Cardiology for the diagnosis and management of patients with ST-elevation myocardial infarction (STEMI) and non-ST-elevation acute coronary syndrome (NSTE-ACS) (including non-STEMI and unstable angina) [7,8].

### Study variables

#### Smoking and smoking cessation interventions

Information on smoking status was obtained from patients’ medical records. Current smoking was defined as smoking within one year preceding the current hospitalization episode [17]. Provision of a smoking cessation intervention before discharge was defined when one of the following conditions was met based on patients’ clinical notes: (a) the patient was given a brochure on smoking cessation; (b) a personalized plan for smoking cessation was made with smokers or their family members; or (c) pharmacotherapy for smoking cessation was prescribed. We did not account for informal oral provision of smoking cessation advice without medical record documentation.

#### Patients’ clinical conditions

Patients’ age of onset of ACS and severe clinical manifestations at admission were recorded. Severe clinical manifestations included STEMI, substantially elevated myocardial injury markers, and critical cardiac symptoms at admission. Critical cardiac symptoms reported at admission included acute heart failure, cardiogenic shock, and cardiac arrest. Acute heart failure, cardiogenic shock, and cardiac arrest at admission were defined based on documentation of patients’ clinical conditions at admission in medical records (Supplementary Methods).

#### In-hospital outcomes

The in-hospital outcomes investigated in this study referred to severe outcomes that occurred during hospitalization, including all-cause mortality, recurrent myocardial infarction, cardiogenic shock, and cardiac arrest.

### Statistical analysis

We first assessed smoking prevalence among male and female ACS patients and further in different subgroups. Categorical variables were presented as percentages and numbers of patients by group and compared using chi-square test or Cochran-Armitage trend test. Smoking prevalence of ACS patients was standardized by age according to the 2010 national census when compared with that in the Chinese general population [18]. We used logistic regression analyses to evaluate the association between smoking status and critical cardiac symptoms at admission and in-hospital outcomes among all ACS patients and in STEMI and NSTE-ACS patients, respectively. All model covariates are presented in Supplementary Table 1, and their definitions are presented in Supplementary Methods. We then compared the proportions of smoking patients receiving cessation interventions and other medications prescribed at discharge.

**Table 1:** Characteristics and pre-hospital and in-hospital treatments of smokers and non-smokers with ACS.

	Smokers (N = 37,750)	Non-smokers (N = 54,759)	*P* value

Age, mean (SD), years	59.1 (11.8)	66.6 (11.9)	<0.001
Women, % (n/N)	5.1 (1,937/37,750)	40.5 (22,201/54,759)	<0.001
Vital signs			
SBP levels, mean (SD), mmHg	128.5 (23.0)	132.2 (23.7)	<0.001
DBP levels, mean (SD), mmHg	78.6 (14.6)	78.1 (14.2)	<0.001
Heart rates, mean (SD), bpm	76.8 (15.8)	78.0 (16.6)	<0.001
Risk factors			
Hypertension, % (n/N)	60.1 (22,703/37,750)	70.1 (38,375/54,759)	<0.001
Diabetes mellitus, % (n/N)	40.0 (15,097/37,750)	47.9 (26,208/54,759)	<0.001
Elevated LDL-C, % (n/N)	54.2 (20,473/37,750)	51.2 (28,029/54,759)	<0.001
Low HDL-C, % (n/N)	49.3 (18,608/37,750)	40.3 (22,086/54,759)	<0.001
Elevated TG, % (n/N)	23.6 (8,902/37,750)	20.0 (1,0957/54,759)	<0.001
History of diseases			
ACS, % (n/N)	9.1 (3,427/37,750)	13.3 (7,299/54,759)	<0.001
Heart failure, % (n/N)	1.1 (398/37,750)	3.1 (1,672/54,759)	<0.001
Atrial fibrillation, % (n/N)	1.3 (501/37,750)	3.2 (1,728/54,759)	<0.001
Cerebrovascular disease, % (n/N)	7.1 (2,685/37,750)	10.4 (5,702/54,759)	<0.001
Family history of CHD, % (n/N)	3.6 (1,341/37,750)	2.1 (1,140/54,759)	<0.001
Critical cardiac symptoms at admission, % (n/N)	8.3 (3,144/37,750)	10.2 (5,576/54,759)	<0.001
Killip class, % (n/N)			<0.001
II–III	20.4 (7,710/37,750)	24.7 (13,509/54,759)	
IV	4.2 (1,600/37,750)	5.0 (2,760/54,759)	
Substantially elevated myocardial injury markers, % (n/N)	86.1 (32,510/37,750)	83.0 (45,468/54,759)	<0.001
ACS type			<0.001
STEMI, % (n/N)	67.8 (25,589/37,750)	54.8 (29,986/54,759)	
NSTE-ACS, % (n/N)	32.2 (12,161/37,750)	45.2 (24,773/54,759)	
Renal insufficiency, % (n/N)	44.6 (16,844/37,750)	61.4 (33,610/54,759)	<0.001
Triple-vessel disease, % (n/N)‖	19.3 (5,534/28,682)	21.1 (7,568/35,875)	<0.001
Pre-hospital statin, % (n/N)	15.2 (5,734/37,750)	19.3 (10,570/54,759)	<0.001
Pre-hospital ACEI/ARB, % (n/N)	8.4 (3,163/37,750)	11.9 (6,533/54,759)	<0.001
Pre-hospital beta-blockers, % (n/N)	7.7 (2,917/37,750)	10.9 (5,956/54,759)	<0.001
Patients with referral, % (n/N)	46.6 (17,581/37,750)	38.0 (20,779/54,759)	<0.001
Medical therapy			
DAPT, % (n/N)	95.2 (35,782/37,588)	89.9 (48,723/54,223)	<0.001
Statins, % (n/N)	94.8 (35,740/37,700)	92.8 (50,709/54,670)	<0.001
ACEI/ARB, % (n/N)	49.8 (17,794/35,726)	50.0 (25,890/51,775)	0.565
Beta-blockers, % (n/N)	58.7 (21,343/36,341)	57.8 (30,508/52,832)	0.003
PCI, % (n/N)	77.9 (29,391/37,750)	64.8 (35,508/54,759)	<0.001


ACEI: angiotensin-converting enzyme inhibitor; ACS: acute coronary syndrome; ARB: angiotensin-receptor blocker; CHD: coronary heart disease; DAPT: dual antiplatelet therapy; DBP: diastolic blood pressure; HDL-C: high-density lipoprotein cholesterol; LDL-C: low-density lipoprotein cholesterol; NSTE-ACS: non-ST-elevation acute coronary syndrome; PCI: percutaneous coronary intervention; SBP: systolic blood pressure; STEMI: ST-elevation myocardial infarction; TG: triglyceride. ‖ Triple-vessel disease was not available for 27,952 (30.2%) patients. The usage rate of drugs was calculated in patients without drug contraindications.

For variables with a missing rate <15%, multiple imputation with chained equations was used to impute missing values by IVEware software version 0.2 (Survey Research Center, University of Michigan, Ann Arbor, MI, USA). Missing rates of variables and strategies for management of missing data are presented in Supplementary Table 2.

Statistical analyses were performed using SAS 9.4 (SAS Institute, Cary, NC, USA). Two-tailed *P* values of <0.05 were considered statistically significant.

## Results

### Smoking prevalence

**Figure 1:**
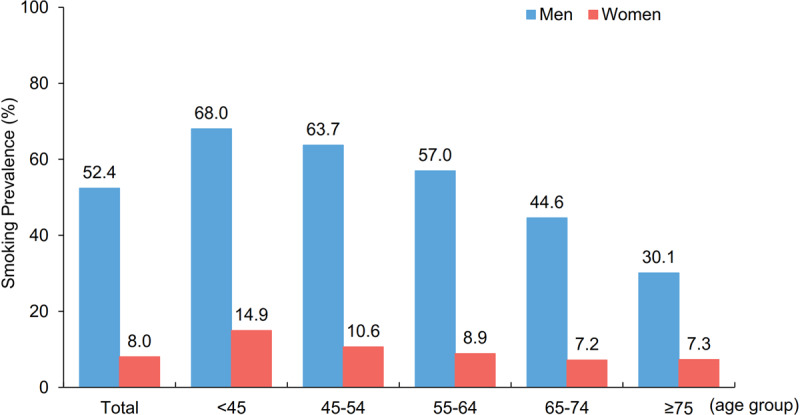
Smoking prevalence among ACS patients by sex and age. Smoking prevalence over the entire study period among different age groups among male ACS patients (blue) and female ACS patients (red). ACS: acute coronary syndrome.

Of our sample of 92,509 ACS patients, 68,371 (73.9%) were men and 24,138 (26.1%) were women. Smoking prevalence was 52.4% in male patients and 8.0% in female patients. Smoking was most common among patients younger than 45 years (men: 68.0%; women: 14.9%) (Figure 1). Among patients with recurrent ACS, 40.2% of male patients and 5.9% of female patients were smokers (Supplementary Table 3). Smoking prevalence was over 50% in 21 of the 30 Chinese provinces among male patients and varied widely between provinces among female patients, from 1.2% in Zhejiang to 26.4% in Tianjin.

The age-standardized smoking prevalence was five times higher in female ACS patients than in Chinese women in general (12.6% versus 2.7%) and 1.3 times higher in male ACS patients than in Chinese men in general (63.0% versus 47.2%) (Supplementary Figure 1).

There was a decline in smoking prevalence among ACS patients from 2014 to 2018, decreasing from 54.6% to 47.3% in male patients and from 9.1% to 6.4% in female patients (Supplementary Figure 2). However, smoking was still the most prevalent risk factor among younger male patients with initial ACS, and smoking prevalence among female patients younger than 45 years increased from 11.8% to 16.0% during the study period.

### Association between smoking and patients’ clinical conditions

#### Onset ages in ACS patients with and without smoking

Among male patients with initial ACS, average onset age in smokers was 58.4 ± 11.7 years, which was 5.9 years younger than that in non-smokers (64.2 ± 12.5 years). In female patients with initial ACS, smokers were 2.1 years younger than non-smokers (67.2 ± 11.4 years versus 69.2 ± 10.6 years). Among male patients with recurrent ACS, average onset age in smokers was 5.7 years younger than in non-smokers (61.7 ± 11.2 years versus 67.4 ± 11.6 years). But there was little difference in average onset age between smokers and non-smokers (72.0 ± 9.9 years versus 71.5 ± 9.9 years) among female patients with recurrent ACS.

#### Association between smoking and severe clinical manifestations

**Figure 2:**
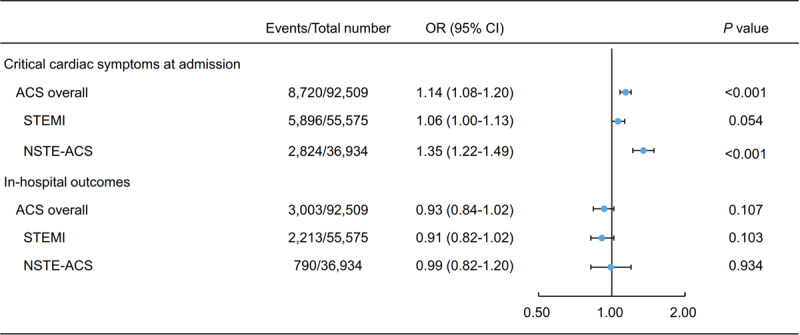
Multivariable analysis of association between smoking and critical cardiac symptoms at admission and in-hospital outcomes among ACS patients. This forest plot shows patients’ critical cardiac symptoms at admission and in-hospital outcomes according to smoking status among all ACS patients, and among patients by subtypes of ACS, using data from the CCC-ACS project. Critical cardiac symptoms at admission included acute heart failure, cardiogenic shock, and cardiac arrest. ACS: acute coronary syndrome; CI: confidence interval; NSTE-ACS: non-ST-elevation acute coronary syndrome; OR: odds ratio; STEMI: ST-elevation myocardial infarction.

Compared with non-smokers, a higher proportion of patients who smoked had STEMI (67.8% versus 54.8%, p < 0.001) and substantially elevated myocardial injury markers (86.1% versus 83.0%, p < 0.001) (Table 1). Smoking was associated with increased odds of critical cardiac symptoms at admission after multivariable adjustment (odds ratio (OR) = 1.14, 95% confidence interval (CI): 1.08–1.20; p < 0.001), especially among NSTE-ACS patients (OR = 1.35, 95% CI: 1.22–1.49; p < 0.001) (Figure 2). The similar findings were identified in both male and female patients. Compared with non-smokers, both male and female patients who smoked had a higher proportion of STEMI (68.0% versus 58.0%, p < 0.001 in men; 63.6% versus 50.0%, p < 0.001 in women) and a higher proportion of the patients with substantially elevated myocardial injury markers (86.1% versus 83.6%, p < 0.001 in men; 87.3% versus 82.2%, p < 0.001 in women). In separated analyses for male and female patients, the association of smoking with higher risk of critical cardiac symptoms at admission remained significant in both male (OR = 1.15, 95% CI: 1.08–1.21; p = 0.004) and female (OR = 1.16, 95% CI: 1.00–1.35; p = 0.048) patients.

### Association between smoking and in-hospital outcomes

Smokers had lower frequency of in-hospital outcomes than non-smokers (2.4% versus 3.9%, p < 0.001), particularly among STEMI patients (2.7% versus 5.1%, p < 0.001) (Supplementary Figure 3). And smokers had lower proportion of in-hospital outcomes than non-smokers in both male (2.3% versus 3.5%, p < 0.001) and female (4.1% versus 4.4%, p = 0.518) patients. However, after adjusting for patients’ demographic characteristics, medical history, clinical manifestations, in-hospital treatment, and other confounding factors, the odds of experiencing the in-hospital outcomes investigated was close to 1 for ACS patients in general (OR = 0.93, 95% CI: 0.84–1.02; p = 0.107), as well as for those with STEMI (OR = 0.91, 95% CI: 0.82–1.02; p = 0.103) or NSTE-ACS (OR = 0.99, 95% CI: 0.82–1.20; p = 0.934) (Figure 2). The association between smoking and in-hospital outcomes remained unchanged in both male (OR = 0.95, 95% CI: 0.85–1.05; p = 0.286) and female (OR = 0.93, 95% CI: 0.72–1.20; p = 0.585) patients.

### Smoking cessation interventions before discharge

Provision of smoking cessation interventions was evaluated in 37,336 smokers with ACS who survived discharge during the study period. Of these patients, 35.3% received smoking cessation interventions before discharge. The proportions of patients receiving smoking cessation interventions varied greatly between hospitals (from 0% to 100%). The rate of provision was <10% in around 40% of hospitals, and overall was inadequate for both male (35.6%) and female (28.7%) smokers. Only 36.0% of smokers younger than 45 years received smoking cessation interventions. Even among smokers with recurrent ACS, only 31.8% received interventions (Supplementary Table 4).

**Figure 3:**
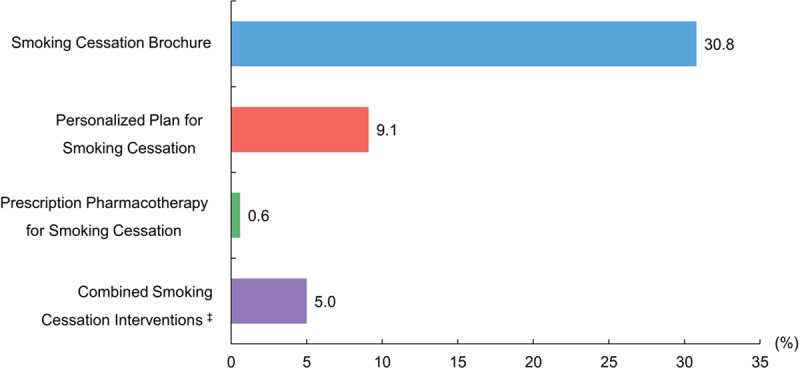
Rates of provision of different types of smoking cessation interventions. Rates of provision of different smoking cessation interventions before discharge for the entire study period among all smokers with ACS who survived to discharge. ^‡^ Provided any two or more above smoking cessation interventions.

Providing smoking cessation brochures to smokers was the most common intervention (30.8%), followed by making personalized cessation plans (9.1%) and prescription of pharmacotherapy (0.6%) (Figure 3).

**Figure 4:**
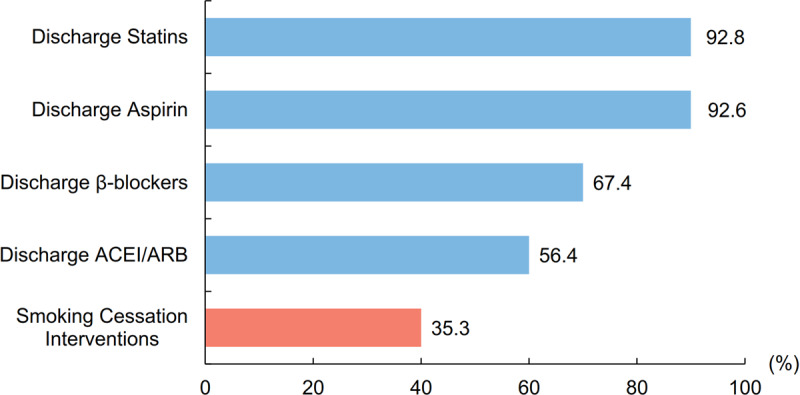
Comparison of patients receiving smoking cessation interventions and medications for secondary prevention before discharge. Proportion of patients receiving smoking cessation interventions and other ACS quality of care measures for the entire study period among all smoking ACS patients before discharge. ACEI: angiotensin-converting enzyme inhibitor; ACS: acute coronary syndrome; ARB: angiotensin-receptor blocker.

We found a promising improvement in provision of smoking cessation interventions, with the yearly rate increasing from 29.4% to 41.9% during the study period (Supplementary Figure 4). However, these proportions were still significantly lower than other quality-of-care indexes, including discharge on aspirin (92.6%), statins (92.8%), beta-blockers (67.4%), and ACEI or angiotensin-receptor blocker (56.4%) (Figure 4).

## Discussion

This study is the largest contemporary registry study to comprehensively evaluate the smoking problem and provision of smoking cessation interventions among ACS patients in China. Our findings are of importance in informing future priorities for improving secondary prevention of ACS in China.

### High smoking prevalence among ACS patients in China

This study demonstrated that smoking was highly prevalent in ACS patients across China. More than half of male patients and around 70% of younger male patients were smokers. Age-standardized smoking prevalence in female patients was five times higher than in Chinese women in general.

Smoking prevalence among ACS patients in China was notably higher than that found in many other countries. The recent European Action on Secondary and Primary Prevention by Intervention to Reduce Events (EUROASPIRE) V survey reported that the mean smoking prevalence among CHD patients was 19.0% in 27 countries [19]. Even as early as 1995, the EUROASPIRE I survey reported a mean smoking prevalence of 19.4% among CHD patients in nine countries [20]. In the United States, the National Cardiovascular Data Registry reported that smoking prevalence among AMI patients was 32.4% in 2017 [21].

Various factors could account for the high smoking prevalence among ACS patients in China. First, smoking among the general Chinese population represents a major public health issue, particularly among men, with overall smoking prevalence significantly exceeding the global average (47.2% versus 25.0%) [22,23]. Second, smoking is a major risk factor for ACS. Previous studies have reported that smokers have a 75% higher risk of ACS than non-smokers [24]. The highly epidemic smoking problem among ACS patients suggests an urgent need to strengthen smoking-related public health interventions in China. Recent population-level tobacco interventions include adopting tobacco clauses (e.g., Advertising Law of 2015) and local smoke-free regulations [22]. While these have been met with declines in smoking prevalence, further efforts are needed to achieve the goal of reducing the nationwide smoking prevalence to 20% by 2030 [25].

### Serious clinical conditions associated with smoking in ACS patients

This study showed that smoking was associated with serious clinical conditions in ACS patients. First, among male patients with initial ACS, smokers experienced ACS approximately six years earlier than non-smokers. Patients in whom ACS occurs in young or middle age have a higher risk of premature mortality and sudden cardiac death outside of hospital. Previous study showed that among younger patients who died of AMI, 90% died outside of hospital [26]. Those patients who survived ACS are more likely to experience a lower quality of life and a limited ability to work [27].

Second, although smokers were younger and had fewer other cardiovascular disease risk factors than non-smoking patients, a higher proportion had a diagnosis of STEMI (67.8% versus 54.8%) and substantially elevated cardiac injury markers (86.1% versus 83.0%) indicating more severe myocardial damage to the heart. Smoking was also associated with an increased risk of critical cardiac symptoms, which are important predictors of poor outcomes at admission [3,5].

### No direct association between smoking and in-hospital outcomes in ACS patients

We did not find that smoking has a direct association with in-hospital outcomes among ACS patients. Previous studies have reported that smokers had lower crude rates of in-hospital outcomes than non-smokers [12,13]. Similar to previous studies, we found that smokers in our study population were younger than non-smokers and less likely to have other cardiovascular risk profiles or experience the in-hospital outcomes investigated. The fact that our multivariable analysis found no difference in in-hospital outcomes between smokers and non-smokers suggests that observed differences of in-hospital outcomes between smokers and non-smokers could be attributable to confounding covariates.

Moreover, after reviewing the studies which reported smokers had lower risk of in-hospital outcomes after ACS than non-smokers [12,13], we found some limitations in their analytical approaches; they did not comprehensively adjust for relevant confounders for the associations between smoking and in-hospital outcomes (e.g., clinical manifestations and in-hospital treatment).

In addition, some studies with longer follow-up periods have found strong associations between smoking and adverse long-term clinical outcomes among ACS patients [28,29,30]. These findings highlight the value of providing smoking cessation interventions for ACS patients.

### Inadequate provision of smoking cessation interventions

We found a low rate of provision of smoking cessation interventions among ACS patients who smoked in this study, with two-thirds of patients not receiving formal intervention before discharge.

Although quitting smoking has been recognized as an effective and cost-effective secondary prevention strategy for CHD patients, the rate of provision of smoking cessation interventions was still much lower than that of other secondary prevention medications. This suggests that smoking cessation interventions remain an underutilized option for secondary ACS prevention in China. Gaps in care might result from inadequate awareness of providers, structural inadequacies of care systems, or physicians’ limited experience in successful provision of smoking cessation interventions. One systematic review found that intensive smoking cessation interventions initiated during hospitalization could help smokers quit smoking, and that the period of hospitalization was a ‘teachable moment’ for patients [9]. Physicians should therefore capitalize on opportunities to provide smoking cessation interventions before discharge for patients who smoke.

High rates of provision of smoking cessation interventions have been achieved in other countries. The EUROASPIRE IV survey reported that around 90% of CHD patients across 24 countries received smoking cessation interventions [31]. Studies from the United States from 2007 to 2017 have reported consistently high rates of provision of smoking cessation interventions for ACS patients (of nearly 100%) [21,32], whereas historically only 40% to 58% of AMI patients were offered smoking cessation interventions [33,34]. This notable increase can be attributed to implementation of quality-improvement programs, which can bridge knowledge gaps or change physicians’ attitudes toward smoking cessation interventions [32]. National tobacco control efforts, including mass media campaigns, tobacco taxes, smoking bans, and plain packaging with graphic warnings, are essential for the achievement of society-wide reductions in smoking prevalence. Although there may be some differences in medical record documentation among different countries, the lower rate of provision of smoking cessation interventions for ACS patients at discharge in China compared with other countries suggested that it remains a significant area for improvement.

### Limitations

This study had some limitations. First, the data about smoking durations and daily numbers of cigarettes of smokers were unavailable and we were unable to study the dose-response association of total smoking exposure with the onset age and in-hospital outcomes among ACS patients. Second, as all patients did not smoke during hospitalization, we were unable to evaluate the effect of smoking cessation during hospitalization on in-hospital outcomes of patients. It is possible that quitting smoking in short term have a potential effect on in-hospital outcomes of patients. Third, this study did not collect information on patients who died before admission. It is possible that smokers with ACS had a greater risk of sudden cardiac death before admission than non-smokers [35], with those who were hospitalized already representing ‘survivors.’

## Conclusions

Smoking is highly prevalent and associated with increased risk of critical cardiac symptoms at admission among ACS patients. However, provision of smoking cessation interventions is very low among ACS patients who smoke, suggesting that there is a substantial gap between guideline-recommended care and clinical practice in China. Implementing formal smoking treatment pathways for ACS patients should be considered a priority for clinical practice in China.

## Data Accessibility Statement

The datasets analyzed during the current study are not publicly available because of intellectual property rights; but are available from the corresponding author on reasonable request.

## Additional File

The additional file for this article can be found as follows:

10.5334/gh.784.s1 Supplementary Files. The additional information of methodology, online figures, and online tables.
