# Supplementary material for: Smoking and Provision of Smoking Cessation Interventions among Inpatients with Acute Coronary Syndrome in China: Findings from the Improving Care for Cardiovascular Disease in China-Acute Coronary Syndrome Project
Source: Glob Heart. 2020 Oct 23;15(1):72. doi: 10.5334/gh.784 (PMC7583717; doi:10.5334/gh.784)
Supplement: Supplementary Files. — The additional information of methodology, online figures, and online tables. [file gh-15-1-784-s1.pdf]

Supplementary Files

Supplementary Methods 2

Supplementary Tables 5

Supplementary Table 1 Adjusted factors for the association between smoking status and critical cardiac symptoms at admission and in-hospital outcomes 5

Supplementary Table 2 Missing rates of each variable and management of missing data (variables without any missing were not list in the following table) 6

Supplementary Table 3 Smoking prevalence of ACS patients by sex 9

Supplementary Table 4 Proportion of receiving smoking cessation interventions among smoking ACS patients...10

Supplementary Table 5 Investigators of CCC-ACS project 11

Supplementary Figures 31

Supplementary Figure 1 Comparison of age-standardized smoking prevalence between ACS patients and the general population by sex 31

Supplementary Figure 2 Trends in smoking prevalence among ACS patients............32

Supplementary Figure 3 In-hospital outcomes of smokers and non-smokers among patients with ACS (overall), STEMI, and NSTE-ACS...33

Supplementary Figure 4 Trends in proportions of patients receiving different smoking cessation interventions...34

Supplementary References..35

Supplementary Methods

Data collection

Data elements collected in this study included patients' demographics, medical history, symptoms on arrival, in-hospital treatments and procedures, discharge medications, and secondary prevention strategies. Eligible patients were consecutively reported to the online data reporting system for each month before the middle of the following month. Data accuracy and completeness are ensured by multiple strategies. Regular on-site quality audits by third-party clinical research associates were performed to ensure data accuracy. Around 5% of reported cases are randomly selected and compared with the original medical records. According to the quality audit reports, the data in this study were appropriately reported with an accuracy rate greater than 95%. The quality audit reports were also fed back to each center regularly to ensure data quality. A standardized online reporting tool with automatic checks for invalid values was used to ensure data completeness. Inconsistent or out-of-range data are checked to prompt the user to correct or review data entries. Also, data quality is monitored monthly and reports are sent to hospitals to ensure data completeness.

Study variable*s*

Definition of acute coronary syndrome (ACS)

ACS, including ST-elevation myocardial infarction (STEMI) and non–ST-elevation acute coronary syndrome (NSTE-ACS), was initially identified based on the principal diagnosis at discharge obtained by reviewing the inpatient list for STEMI, non-ST-elevation myocardial infarction and unstable angina. The diagnostic criteria for ACS were based on chest pain,

ECG, and measurements of myocardial injury biomarkers, and were consistent with the definitions of STEMI and NSTE-ACS in the American College of Cardiology/American Heart Association (ACC/AHA) guidelines and the European Society of Cardiology (ESC) guidelines [1-4].

Definition of other variables

Patients with hypertension were defined as those with a prior history of hypertension, receiving antihypertensive therapy, or with a systolic blood pressure of ≥140 mmHg or diastolic blood pressure of ≥90 mmHg on admission. Patients with diabetes mellitus were defined as those having a previous or new diagnosis of diabetes mellitus, receiving anti-diabetic treatment, or having a fasting blood glucose level of ≥7.0 mmol/L (126 mg/dL) or hemoglobin A1c level of ≥6.5%. Patients with elevated low-density lipoprotein cholesterol (LDL-C) were those with a serum LDL-C level of ≥2.6 mmol/L (100 mg/dL). The cutoff for low high-density lipoprotein cholesterol (HDL-C) was defined as a serum HDL-C concentration of <1.0 mmol/L (40 mg/dL). Elevated triglyceride (TG) was defined as a serum TG concentration of ≥2.3 mmol/L (200mg/dl). Estimated glomerular filtration rate (eGFR) was calculated using the equation developed by the Chronic Kidney Disease Epidemiology Collaboration [5]. Patients with renal insufficiency were defined as those with eGFR <90 mL/min per 1.73 m^2 and a history of renal failure. A history of ACS was specified if the patient had a history of myocardial infarction or had undergone percutaneous coronary intervention or coronary artery bypass grafting before the current hospitalization episode. Patient history of other conditions, including heart failure, atrial fibrillation, cerebrovascular disease, renal failure, and family history of coronary heart disease was defined according to

notes obtained from patients' original medical records. The definition of substantially elevated myocardial injury markers was elevation of cardiac injury markers beyond five times the upper reference limit [4]. Patients with triple-vessel disease were defined as those with stenosis of ≥50% in all three main epicardial coronary arteries, including left anterior descending, left circumflex, and right coronary artery (diagnosed by angiography). According to the Chinese Guidelines for the Diagnosis and Treatment of Heart Failure 2014 [6], patients with Killip class II-III were classified as acute heart failure and patients with Killip class IV were classified as cardiogenic shock in clinical practice. Patients' transfer status indicated whether they had been transferred from another hospital.

Statistical analysis

We compared the characteristics of smokers and non-smokers. Categorical variables were presented as percentage of the total sample (and numbers of patients) by group and differences between groups were compared using Pearson's chi-square test. Means (and standard deviations) were presented for continuous variables with normal distributions, and differences between groups were compared using t-tests.

Supplementary Tables

Supplementary Table 1 Adjusted factors for the association between smoking status and critical cardiac symptoms at admission and in-hospital outcomes

	Adjusted factors
Critical cardiac symptoms at admission	Age, sex, smoke, hypertension, diabetes mellitus, heart rate, history of ACS, history of heart failure, history of atrial fibrillation, history of cerebrovascular disease, history of renal failure, pre-hospital statin treatment, pre-hospital ACEI/ARB treatment, pre-hospital beta-blocker treatment, type of ACS
In-hospital outcomes	Age, sex, smoke, diabetes mellitus, elevated LDL-C, systolic blood pressure, heart rate, history of ACS, history of heart failure, history of cerebrovascular disease, type of ACS, renal insufficiency, triple-vessel disease, cardiac arrest at admission, substantially elevated myocardial injury markers and Killip class at admission, DAPT at arrival, statins at arrival, ACEI/ARB at arrival, beta-blocker at arrival, PCI, hospital level, transfer status

ACEI: angiotensin-converting enzyme inhibitor; ACS: acute coronary syndrome; ARB: angiotensin-receptor blocker; DAPT: dual antiplatelet therapy; LDL-C: low-density lipoprotein cholesterol; PCI: percutaneous coronary intervention

Supplementary Table 2 Missing rates of each variable and management of missing data (variables without any missing were not list in the following table)

Variables	Missing, N (%)		Management of missing data
	STEMI (n=55,575)	NSTE-ACS (n=36,934)	
Age	184 (0.33%)	148 (0.40%)	Patients with missing values were imputed using multiple imputation with chained equations implemented by IVEware software.
Sex	3 (0.01%)	2 (0.01%)	
Systolic blood pressure	117 (0.21%)	43 (0.12%)	
Diastolic blood pressure	133 (0.24%)	64 (0.17%)	
Heart rate	146 (0.26%)	73 (0.20%)	
LDL-C	5,957 (10.72%)	3,534 (9.57%)	
HDL-C	5,789 (10.42%)	3,445 (9.33%)	
TG	5,565 (10.01%)	3,390 (9.18%)	
Serum creatinine	1,969 (3.54%)	1,498 (4.06%)	
Fasting blood glucose	7,126 (12.82%)	4,908 (13.29%)	

Cardiac arrest at admission	555 (1.00%)	274 (0.74%)	
Substantially elevated myocardial injury markers	1,058 (1.90%)	959 (2.60%)	
Acute heart failure at admission	538 (0.97%)	250 (0.68%)	Patients with unclear status for acute heart failure and cardiogenic shock were imputed by Killip class. Patients with Killip class II-III were classified as acute heart failure and patients with Killip class IV were classified as cardiogenic shock. If patients with unclear status of Killip class, we imputed the missing values using multiple imputation with chained equations implemented by IVEware software.
Cardiogenic shock at admission	515 (0.93%)	248 (0.67%)	
Killip class	4180 (7.52%)	9353 (25.32%)	Patients with unclear status for Killip class were imputed based on the status of acute heart failure or cardiogenic shock at admission. Patients with acute heart failure were identified as Killip class II-III and patients with cardiogenic shock were identified as Killip class IV. Patients without both of acute heart failure and cardiogenic shock were identified as Killip class I. If patients with

	unclear status of acute heart failure or cardiogenic shock, we imputed the missing values using multiple imputation with chained equations implemented by IVEware software.

HDL-C: high-density lipoprotein cholesterol; LDL-C: low-density lipoprotein cholesterol; NSTE-ACS: non-ST-elevation acute coronary syndrome; STEMI: ST-elevation myocardial infarction; TG: triglyceride

Supplementary Table 3 Smoking prevalence of ACS patients by sex

	Men (N=68,371)	Women (N=24,138)
Overall, % (n/N)	52.4 (35,813/68,371)	8.0 (1,937/24,138)
Age (years), % (n/N)		
<45	68.0 (4,310/6,336)	14.9 (57/382)
45-54	63.7 (9,631/15,114)	10.6 (219/2,062)
55-64	57.0 (11,405/20,023)	8.9 (508/5,724)
65-74	44.6 (7,294/16,362)	7.2 (580/8,112)
≥75	30.1 (3,173/10,536)	7.3 (573/7,858)
History of ACS, % (n/N)		
Yes	40.2 (3,276/8,153)	5.9 (151/2,573)
No	54.0 (32,537/60,218)	8.3 (1,786/21,565)

ACS: acute coronary syndrome

Supplementary Table 4 Proportion of receiving smoking cessation interventions among smoking ACS patients

	Total (N=37,336)	Men (N=35,441)	Women (N=1,895)
Overall, % (n/N)	35.3 (13,161/37,336)	35.6 (12,617/35,441)	28.7 (544/1,895)
Age (years), % (n/N)			
<45	36.0 (1,566/4,349)	36.1 (1,550/4,292)	28.1 (16/57)
45-54	35.4 (3,464/9,796)	35.5 (3,401/9,579)	29.0 (63/217)
55-64	35.3 (4,173/11,825)	35.7 (4,042/11,321)	26.0 (131/504)
65-74	35.4 (2,738/7,741)	35.7 (2,562/7,171)	30.9 (176/570)
≥75	33.7 (1,220/3,625)	34.5 (1,062/3,078)	28.9 (158/547)
History of ACS, % (n/N)			
Yes	31.8 (1,071/3,372)	32.0 (1,032/3,226)	26.7 (39/146)
No	35.6 (12,090/33,964)	36.0 (11,585/32,215)	28.9 (505/1,749)
Types of ACS, % (n/N)			
STEMI	35.5 (8,978/25,268)	35.9 (8,648/24,063)	27.4 (330/1,205)
NSTE-ACS	34.7 (4,183/12,068)	34.9 (3,969/11,378)	31.0 (214/690)

ACS: acute coronary syndrome; NSTE-ACS: non-ST-elevation acute coronary syndrome; STEMI: ST-elevation myocardial infarction

Supplementary Table 5 Investigators of CCC-ACS project

ID	Hospitals	Territories	Provinces	City	Investigator
1	Shanxi Cardiovascular Hospital	Northern China	Shanxi	Taiyuan	Bao Li
2	Nanjing Drum Tower Hospital, The Affiliated Hospital of Nanjing University Medical School	Eastern China	Jiangsu	Nanjing	Biao Xu
3	Hainan General Hospital	Southern China	Hainan	Haikou	Bin Li
4	The Second Hospital of Jilin University	Northeast China	Jilin	Changchun	Bin Liu
5	The 2nd Affiliated Hospital of Harbin Medical University	Northeast China	Heilongjiang	Harbin	Bo Yu
6	The Ninth Hospital Affiliated to Shanghai Jiaotong University School of Medicine	Eastern China	Shanghai	Shanghai	Changqian Wang
7	Henan Provincial People's Hospital	Central China	Henan	Zhengzhou	Chuanyu Gao
8	Shanxi Provincial People's Hospital	Northern China	Shanxi	Taiyuan	Chunlin Lai
9	Xinqiao Hospital, Third Military Medical University	Southwest China	Chongqing	Chongqing	Cui Bin, Lan Huang
10	China Meitan General Hospital	Northern China	Beijing	Beijing	Di Wu

11	The 309th Hospital of Chinese People's Liberation Army	Northern China	Beijing	Beijing	Fakuan Tang, Jun Xiao
12	Zhongda Hospital, Southeast University	Eastern China	Jiangsu	Nanjing	Genshan Ma
13	The First Affiliated Hospital of Liaoning Medical University	Northeast China	Liaoning	Jinzhou	Guizhou Tao
14	Xinjiang Uygur Autonomous Region People's Hospital	Northwest China	Xinjiang	Urumchi	Guoqing Li
15	Sir Run Run Shaw Hospital, College of Medicine, Zhejiang University	Eastern China	Zhejiang	Hangzhou	Guosheng Fu
16	Beijing Friendship Hospital, Capital Medical University	Northern China	Beijing	Beijing	Hongwei Li
17	The First Affiliated Hospital of Bengbu Medical College	Eastern China	Anhui	Bengbu	Hongju Wang, Bi Tang
18	General Hospital of TISCO	Northern China	Shanxi	Taiyuan	Huifeng Wang
19	Dongguan People's Hospital	Southern China	Guangdong	Dongguan	Jianfeng Ye
20	Panyu Hospital of Chinese Medicine	Southern China	Guangdong	Guangzhou	Jianhao Li

21	Peking University First Hospital	Northern China	Beijing	Beijing	Jie Jiang
22	Sun Yat-sen Memorial Hospital, Sun Yat-sen University	Southern China	Guangdong	Guangzhou	Jingfeng Wang
23	Guangdong General Hospital	Southern China	Guangdong	Guangzhou	Jiyan Chen
24	Hospital of Xinjiang Production & Construction Corps	Northwest China	Xinjiang	Urumchi	Junming Liu
25	The Military General Hospital of Beijing PLA	Northern China	Beijing	Beijing	Junxia Li
26	The First Affiliated Hospital of Guangxi Medical University	Southern China	Guangxi	Nanning	Lang Li
27	Tongren Hospital Affiliated to Shanghai Jiaotong University School of Medicine	Eastern China	Shanghai	Shanghai	Li Jiang
28	Binzou City Center Hospital	Eastern China	Shandong	Binzhou	Lijun Meng, Junqiang Cao
29	The First Affiliated Hospital of Zhengzhou University	Central China	Henan	Zhengzhou	Ling Li
30	Xijing Hospital	Northwest China	Shaanxi	Xi’an	Ling Tao
31	The Affiliated Hospital of Guizhou Medical University	Southwest China	Guizhou	Guiyang	Lirong Wu

32	First Affiliated Hospital of the People's Liberation Army General Hospital	Northern China	Beijing	Beijing	Miao Tian
33	The Second People's Hospital of Yunnan Province	Southwest China	Yunnan	Kunming	Minghua Han
34	Haikou People's Hospital	Southern China	Hainan	Haikou	Moshui Chen
35	Gansu Provincial Hospital	Northwest China	Gansu	Lanzhou	Ping Xie
36	The First Affiliated Hospital of Henan University of Science and Technology	Central China	Henan	Luoyang	Pingshuan Dong, Xuming Yang
37	Chenzhou First People's Hospital	Central China	Hunan	Chenzhou	Qiaoqing Zhong, Dajun Hu
38	People's Hospital of Qinghai Province	Northwest China	Qinghai	Xining	Rong Chang, Xiaoling Su
39	Affiliated Hospital of Ningxia Medical University	Northwest China	Ningxia	Yinchuan	Shaobin Jia
40	Beijing Anzhen Hospital, Capital Medical University	Northern China	Beijing	Beijing	Shaoping Nie, Xiaohui Liu,

					Qiang Lv
41	North Jiangsu People’s Hospital	Eastern China	Jiangsu	Yangzhou	Shenghu He
42	Shanghai Sixth People’s Hospital	Eastern China	Shanghai	Shanghai	Shixin Ma
43	The First Hospital of Handan	Northern China	Hebei	Handan	Shuanli Xin
44	Huai’an First People’s Hospital	Eastern China	Jiangsu	Huai’an	Shuren Ma
45	The First Affiliated Hospital of Chongqing Medical University	Southwest China	Chongqing	Chongqing	Suxin Luo
46	Navy General Hospital	Northern China	Beijing	Beijing	Tianchang Li
47	Zhejiang Provincial Hospital of TCM	Eastern China	Zhejiang	Hangzhou	Wei Mao
48	The Third Xiangya Hospital of Central South University	Central China	Hunan	Changsha	Weihong Jiang
49	Affiliated Hospital of Qinghai University	Northwest China	Qinghai	Xining	Weijun Liu
50	Teda International Cardiovascular Hospital	Northern China	Tianjin	Tianjin	Wenhua Lin
51	The Second Hospital of Hebei Medical University	Northern China	Hebei	Shijiazhuang	Xianghua Fu
52	Changhai Hospital of Shanghai	Eastern China	Shanghai	Shanghai	Xianxian Zhao

53	The Second Affiliated Hospital to Nanchang University	Eastern China	Jiangxi	Nanchang	Xiaoshu Cheng
54	Hebei General Hospital	Northern China	Hebei	Shijiazhuang	Xiaoyong Qi
55	Inner Mongolia People’s Hospital	Northern China	Inner Mongolia	Hohhot	Xingsheng Zhao
56	The General Hospital of Shenyang Military Region	Northeast China	Liaoning	Shenyang	Yaling Han
57	The First Hospital of Jilin University	Northeast China	Jilin	Changchun	Yang Zheng
58	Tianjin Chest Hospital	Northern China	Tianjin	Tianjin	Yin Liu
59	Hunan Provincial People’s Hospital	Central China	Hunan	Changsha	Ying Guo, Jianqiang Peng
60	People’s Hospital of Yuxi City	Southwest China	Yunnan	Yuxi	Yinglu Hao
61	The People’s Hospital of Guangxi Zhuang Autonomous Region	Southern China	Guangxi	Nanning	Yingzhong Lin
62	The First Teaching Hospital of Xinjiang Medical University	Northwest China	Xinjiang	Urumchi	Yitong Ma

63	Baogang Hospital	Northern China	Inner Mongolia	Baotou	Yongdong Li
64	Tianjin Medical University General Hospital	Northern China	Tianjin	Tianjin	Yuemin Sun
65	The Second Affiliated Hospital of Zhengzhou University	Central China	Henan	Zhengzhou	Yulan Zhao
66	Nanfang Hospital of Southern Medical University	Southern China	Guangdong	Guangzhou	Yuqing Hou
67	The First Affiliated Hospital to Nanchang University	Eastern China	Jiangxi	Nanchang	Zeqi Zheng
68	The First Affiliated Hospital of Lanzhou University	Northwest China	Gansu	Lanzhou	Zheng Zhang
69	The Third Hospital of Shijiazhuang	Northern China	Hebei	Shijiazhuang	Zhenguo Ji
70	Wuxi People’s Hospital	Eastern China	Jiangsu	Wuxi	Zhenyu Yang
71	Jiangsu Province Hospital	Eastern China	Jiangsu	Nanjing	Zhijian Yang
72	The Second Hospital of Shanxi Medical University	Northern China	Shanxi	Taiyuan	Zhiming Yang
73	The Affiliated Hospital of Xuzhou Medical College	Eastern China	Jiangsu	Xuzhou	Zhirong Wang
74	Southwest Hospital, Third Military Medical University	Southwest China	Chongqing	Chongqing	Zhiyuan Song
75	The First Affiliated Hospital of Xi’an Jiaotong University	Northwest China	Shaanxi	Xi’an	Zuyi Yuan

76	Yangzhou First People’s Hospital	Eastern China	Jiangsu	Yangzhou	Aihua Li
77	Hospital 463 of Chinese People’s Liberation Army	Northeast China	Liaoning	Shenyang	Bosong Yang
78	The Central Hospital of Mianyang	Northwest China	Sichuan	Mianyang	Caidong Luo
79	Liaocheng People’s Hospital	Eastern China	Shandong	Liaocheng	Chunyan Zhang
80	Yancheng Third People’s Hospital	Eastern China	Jiangsu	Yancheng	Chunyang Wu
81	The Second Xiangya Hospital of Central South University	Central China	Hunan	Changsha	Daoquan Peng
82	The Central Hospital of Panzhihua	Northwest China	Sichuan	Panzhihua	Dawen Xu
83	The First Hospital of Qiqihar City	Northeast China	Heilongjiang	Qiqihar	Gang Xu
84	The Third the People’s Hospital of Bengbu	Eastern China	Anhui	Bengbu	Gengsheng Sang
85	The First Hospital of Jiamusi	Northeast China	Heilongjiang	Jiamusi	Guixia Zhang
86	Zhoushan People’s Hospital	Eastern China	Zhejiang	Zhoushan	Guoxiong Chen
87	Dalian Municipal Central Hospital	Northeast China	Liaoning	Dalian	Hailong Lin
88	Renmin Hospital of Wuhan University	Central China	Hubei	Wuhan	Hong Jiang
89	Ningxia People’s Hospital	Northwest China	Ningxia	Yinchuan	Hong Luan

90	The First People's Hospital of Yunnan Province (Kunhua Hospital)	Southwest China	Yunnan	Kunming	Hong Zhang
91	The Central Hospital of Zhoukou	Central China	Henan	Zhoukou	Hualing Liu
92	Anyang District Hospital	Central China	Henan	Anyang	Hui Liu
93	Sichuan Provincial People's Hospital	Northwest China	Sichuan	Chengdu	Jianhong Tao
94	Mudanjiang Cardiovascular Disease Hospital	Northeast China	Heilongjiang	Mudanjiang	Jianwen Liu
95	Yichang Central Hospital	Central China	Hubei	Yichang	Jiawang Ding
96	Qilu Hospital of Shandong University	Eastern China	Shandong	Jinan	Jifu Li
97	Affiliated Hospital of Jiangsu University	Eastern China	Jiangsu	Zhenjiang	Jinchuan Yan
98	The First People's Hospital of Nanning City	Southern China	Guangxi	Nanning	Jinru Wei
99	The First Affiliated Hospital of Fujian Medical University	Eastern China	Fujian	Fuzhou	Jinzi Su
100	Chengdu Third People's Hospital	Northwest China	Sichuan	Chengdu	Jiong Tang
101	Yantaishan hospital	Eastern China	Shandong	Yantai	Juexin Fan
102	Qingdao Municipal Hospital	Eastern China	Shandong	Qingdao	Jun Guan

103	Zhongshan Hospital Affiliated to Fudan University	Eastern China	Shanghai	Shanghai	Junbo Ge
104	Longyan First Hospital	Eastern China	Fujian	Longyan	Kaihong Chen
105	Affiliated Hospital of Guangdong Medical College	Southern China	Guangdong	Guangzhou	Keng Wu
106	Jiangxi Provincial People's Hospital	Eastern China	Jiangxi	Nanchang	Lang Ji
107	Anhui Provincial Hospital	Eastern China	Anhui	Hefei	Likun Ma
108	Xiangtan City Central Hospital	Central China	Hunan	Xiangtan	Lilong Tang
109	The First Hospital of Haerbin City	Northeast China	Heilongjiang	Harbin	Lin Wei
110	Central Hospital Affiliated to Shenyang Medical College	Northeast China	Liaoning	Shenyang	Man Zhang
111	The Central Hospital of Wuhan	Central China	Hubei	Wuhan	Manhua Chen
112	Hangzhou First People's Hospital	Eastern China	Zhejiang	Hangzhou	Ningfu Wang
113	The Central Hospital of Xuzhou	Eastern China	Jiangsu	Xuzhou	Bing Han
114	The Second hospital of Dalian Medical University	Northeast China	Liaoning	Dalian	Peng Qu
115	The First Affiliated Hospital of Liaoning University of Traditional Chinese Medicine	Northeast China	Liaoning	Shenyang	Ping Hou

116	Beijing Tsinghua Changgung Hospital	Northern China	Beijing	Beijing	Ping Zhang
117	Guizhou Provincial People's Hospital	Southwest China	Guizhou	Guiyang	Qiang Wu
118	The First Affiliated Hospital of Xiamen University	Eastern China	Fujian	Xiamen	Qiang Xie
119	Quanzhou First Hospital	Eastern China	Fujian	Quanzhou	Rong Lin
120	Wuzhou People's Hospital	Southern China	Guangxi	Wuzhou	Shaowu Ye, Zhaohua Lu
121	The Central Hospital of Jilin	Northeast China	Jilin	Changchun	Shuangbin Li
122	Xiangya Hospital Central South University	Central China	Hunan	Changsha	Tianlun Yang
123	Guangzhou Red Cross Hospital	Southern China	Guangdong	Guangzhou	Tongguo Wu
124	The First Affiliated Hospital of Guangzhou Medical College	Southern China	Guangdong	Guangzhou	Wei Wang
125	The First Affiliated Hospital of Wenzhou Medical University	Eastern China	Zhejiang	Wenzhou	Weijian Huang
126	The Second Affiliated Hospital of Soochow University	Eastern China	Jiangsu	Suzhou	Weiting Xu

127	Wuhan Asia Heart Hospital	Central China	Hubei	Wuhan	Xi Su
128	The First Affiliated Hospital of Soochow University	Eastern China	Jiangsu	Suzhou	Xiangjun Yang
129	Affiliated Hospital of Yan'an University	Northwest China	Shaanxi	Yan'an	Xiaochuan Ma
130	The First People's Hospital of Jining	Eastern China	Shandong	Jining	Xiaofei Sun
131	The Central Hospital of Taiyuan	Northern China	Shanxi	Taiyuan	Xiaoping Chen
132	West China Hospital of Sichuan University	Northwest China	Sichuan	Chengdu	Xiaoping Chen
133	The Third Affiliated Hospital of Guangzhou Medical College	Southern China	Guangdong	Guangzhou	Ximing Chen
134	The First Affiliated Hospital of Wannan Medical College	Eastern China	Anhui	Wuhu	Xingsheng Tang
135	Tangdu Hospital of The Fourth Military Medical University	Northwest China	Shaanxi	Xi'an	Xue Li
136	Shanghai East Hospital Affiliated to Tongji University	Eastern China	Shanghai	Shanghai	Xuebo Liu
137	Xiamen Cardiovascular Disease Hospital	Eastern China	Fujian	Xiamen	Yan Wang
138	Zhongnan hospital of Wuhan University	Central China	Hubei	Wuhan	Yanggan Wang

139	Fujian Provincial Hospital	Eastern China	Fujian	Fuzhou	Yansong Guo
140	The First Affiliated hospital of Dalian Medical University	Northeast China	Liaoning	Dalian	Yanzong Yang
141	The First People's Hospital of Changde	Central China	Hunan	Changde	Yi Huang,<br>Liangqing Ge
142	The First Affiliated Hospital of China Medical University	Northeast China	Liaoning	Shenyang	Yingxian Sun
143	The Fourth Affiliated Hospital of China Medical University	Northeast China	Liaoning	Shenyang	Yuanzhe Jin
144	Cangzhou Central Hospital	Northern China	Hebei	Cangzhou	Zesheng Xu
145	The Central Hospital of Shaoyang	Central China	Hunan	Shaoyang	Zewei Ouyang
146	The People's Hospital of Liaoning Province	Northeast China	Liaoning	Shenyang	Zhanquan Li
147	The First Affiliated Hospital of Jiamusi University	Northeast China	Heilongjiang	Jiamusi	Zhaofa He
148	Tangshan Gongren Hospital	Northern China	Hebei	Tangshan	Zheng Ji
149	Huaibei Miners General Hospital	Eastern China	Anhui	Huaibei	Zhenqi Su
150	Linyi People's Hospital	Eastern China	Shandong	Linyi	Zhihong Ou

151	Chongqing Hechuan District People’s Hospital	Southwest China	Chongqing	Chongqing	Xin Tang
152	Yuzhou City Central Hospital	Central China	Henan	Xuchang	Qinfeng Su
153	Jianshui County People’s Hospital	Southwest China	Yunnan	Honghe	Weiqing Fan
154	Dunhua City Hospital	Northeast China	Jilin	Dunhua	Fanju Meng
155	Shenyang City Electricity Central Hospital	Northeast China	Liaoning	Shenyang	Jing Xu
156	Shanghai Jingan District Shibei Hospital	Eastern China	Shanghai	Shanghai	Bin Wang
157	Beijing Fangshan District First Hospital	Northern China	Beijing	Beijing	Xuemei Peng
158	Hebei Daming County People’s Hospital	Northern China	Hebei	Handan	Haiping Guo
159	Jiangsu Binhai County People’s Hospital	Eastern China	Jiangsu	Yancheng	Yonglin Zhang
160	The First People’s Hospital of Longquanyi District	Southwest China	Sichuan	Chengdu	Wei Tuo
161	Guangxi Hengxian County People’s Hospital	Southern China	Guangxi	Nanning	Xianan Zhang
162	Hunan Changsha County First People’s Hospital	Central China	Hunan	Changsha	Siding Wang
163	People’s Hospital of Wugang	Central China	Hunan	Shaoyang	JiaoMei Yang
164	Longhui County People’s Hospital	Central China	Hunan	Shaoyang	Xiaojun Wang

165	Heilongjiang Fujin City Central Hospital	Northeast China	Heilongjiang	Jiamusi	Jiyan Yin
166	Dalian Fourth People’s Hospital	Northeast China	Liaoning	Dalian	Huifang Zhang
167	General Hospital of Guangzhou Military Command	Southern China	Guangdong	Guangzhou	Yanlie Zheng
168	The First People’s Hospital of Horqin District, Tongliao City	Northern China	Inner Mongolia	Tongliao	Junping Fang
169	Guiyang Sixth People’s Hospital	Southwest China	Guizhou	Guiyang	Kalan Luo
170	Geological Mining Hospital of Hunan Province	Central China	Hunan	Changsha	Naiyi Liang
171	Zhangzhou Municipal Hospital of Fujian Province	Eastern China	Fujian	Zhangzhou	Changyong Liu
172	Jining City Yanzhou District People’s Hospital	Eastern China	Shandong	Jining	Jian Yang
173	The People’s Hospital Feixian	Eastern China	Shandong	Linyi	Honghua Deng
174	Tangshan City Fengrun District People’s Hospital	Northern China	Hebei	Tangshan	Lin Wang
175	Qian’an People’s Hospital	Northern China	Hebei	Tangshan	Yuheng Yang
176	Yuzhong County People’s Hospital	Northwest China	Gansu	Lanzhou	Xiaowei Peng
177	Baiyin Cite Center Hospital	Northwest China	Gansu	Baiyin	Fang Zhao

178	Mingguang People's Hospital	Eastern China	Anhui	Chuzhou	Yong Li
179	Xihua County People's Hospital	Central China	Henan	Zhoukou	Chuntong Wang
180	Zhalantun People's Hospital	Northern China	Inner Mongolia	Hulunbeier	Yuhua Zhu
181	Fengrun District Second People's Hospital	Northern China	Hebei	Tangshan	Jingshan Zhao, Hua Jiang
182	Zhangping City Hospital	Eastern China	Fujian	Zhangpin	Jinxing Yi
183	Fuqing Cite Hospital	Eastern China	Fujian	Fuqing	Ping Chen
184	The Eight Affiliated Hospital, Sun Yat-sen University	Southern China	Guangdong	Guangzhou	Nan Jia
185	The Second Affiliated Hospital of Qiqihar Medical University	Northeast China	Heilongjiang	Qiqihar	Yanli Wang
186	Wuhan University of Science and Technology Hospital	Central China	Hubei	Wuhan	Jing Hu
187	Baotou City Center Hospital	Northern China	Inner Mongolia	Baotou	Ruiping Zhao

188	Shanghai Jiading District Center Hospital	Eastern China	Shanghai	Shanghai	Xia Chen
189	Datong City Second People’s Hospital	Northern China	Shanxi	Datong	Xiaoqin Zhang
190	Binyang People’s Hospital	Southern China	Guangxi	Binyang	Fudong Gan
191	Deqing People’s Hospital	Eastern China	Zhejiang	Deqing	Fangfang Huang
192	Xinmi people’s hospital	Central China	Henan	Xinmi	Xiaolei Li
193	Dongguan Changping hospital	Southern China	Guangdong	Dongguan	Haiyun Lin
194	Gongyi people’s hospital	Central China	Henan	Gongyi	Tianmin Du
195	Ye County people’s hospital	Central China	Henan	Yexian	Jie Yang
196	The second people’s hospital of Mengcheng	Eastern China	Anhui	Mengcheng	Pengfei Zhang
197	Nanpi People’s Hospital	Northern China	Hebei	Nanpi	Hui Dong
198	Shimen People’s Hospital	Central China	Hunan	Shimeng	Chuanliang Liang
199	Tieli People’s Hospital	Northeast China	Heilongjiang	Tieli	Yanbo Niu
200	Sihui People’s Hospital	Southern China	Guangdong	Sihui	Yuehua Huang
201	Chest Hospital of Xinjiang Uygur Autonomous Region	Northwest China	Xinjiang	Urumchi	Dongsheng Chai

202	Beian First People’s Hospital	Northeast China	Heilongjiang	Bei’an	Dongyan Li
203	Zunhua People’s Hospital	Northern China	Hebei	Zunhua	Xiaoli Yang
204	Lujiang People’s Hospital	Eastern China	Anhui	Lujiang	Qichun Wang
205	Qinyang People’s Hospital	Central China	Henan	Qinyang	Xiaowen Ma
206	Longmen People’s Hospital	Southern China	Guangdong	Longmen	Yingchao Luo
207	Quyang Renji Hospital	Northern China	Hebei	Quyang	Congliang Zhang
208	Nenjiang People’s Hospital	Northeast China	Heilongjiang	Nenjiang	Shuhua Zhang
209	Longjiang First People’s Hospital	Northeast China	Heilongjiang	Longjiang	Yuhuan Shi
210	Li County Hospital of Traditional Chinese Medicine	Central China	Hunan	Changde	Songbai Li
211	Luan County People’s Hospital	Northern China	Hebei	Luanxian	Guo Li
212	Yulong Hospital	Southwest China	Yunnan	Yulong	Zeyuan He
213	Huining People’s Hospital	Northwest China	Gansu	Huining	Jiabin Xi
214	Yuncheng Hospital	Eastern China	Shandong	Yuncheng	Jinglan Diao
215	Hepu People’s Hospital	Southern China	Guangxi	Hepu	Meisheng Lai

216	Duzishan Petrochemical Hospital	Northwest China	Xinjiang	Dushanzi	Shuqiu Qu
217	Guiding People’s Hospital	Southwest China	Guizhou	Guiding	Guoduo Chen
218	People’s Hospital of Rongchang District	Southwest China	Chongqing	Chongqing	Jie Chen
219	Ningbo First Hospital	Eastern China	Zhejiang	Ningbo	Huimin Chu
220	Ledong Second People’s Hospital	Southern China	Hainan	Ledong	Xiufeng Chen
221	Guang’an People’s Hospital	Southwest China	Sichuan	Guang’an	Tian Tuo
222	Linfen People’s Hospital	Northern China	Shanxi	Linfen	Junping Deng
223	People’s Hospital of Bozhou District	Southwest China	Guizhou	Zunyi	Shengyong Chen
224	Dianjiang People’s Hospital	Southwest China	Chongqing	Dianjiang	Yang Yu
225	First Affiliated Hospital of Harbin Medical University.	Northeast China	Heilongjiang	Harbin	Yue Li
226	Yiliang Hospital	Southwest China	Yunnan	Yiliang	Liqiong Yang
227	Haidong Ping’an District Hospital of Traditional Chinese Medicine	Northwest China	Qinghai	Haidong	Guoqin Xin
228	Ningjin People’s Hospital	Eastern China	Shandong	Ningjin	Tao Zhang

229	Yutian Hospital	Northern China	Hebei	Yutian	Xiaoyun Feng
230	Yanting People's Hospital	Southwest China	Sichuan	Yanting	Mingcheng Bai
231	The Fourth Affiliated Hospital Zhejiang University School of Medicine	Eastern China	Zhejiang	Yiwu	Shudong Xia
232	Wuxi Xishan People's Hospital	Eastern China	Jiangsu	Wuxi	Xudong Li
233	Dongfeng Hospital	Northeast China	Jilin	Dongfeng	Wei Liu
234	Zhijin People's Hospital	Southwest China	Guizhou	Zhijin	Zhongshan Wang
235	Huaiyang People's Hospital	Central China	Henan	Huaiyang	Li Wei
236	Suizhou Central Hospital	Central China	Hubei	Suizhou	Fengwei Li
237	Tonglu First People's Hospital	Eastern China	Zhejiang	Tonglu	Xiaolan Li
238	Xiantao First People's Hospital	Central China	Hubei	Xiantao	Dongmei Zhu
239	Honghu People's Hospital	Central China	Hubei	Honghu	Hong Liu
240	Xinjin County Hospital of Traditional Chinese Medicine	Northwest China	Sichuan	Xinjin	Yingbi Su

CCC-ACS: The Improving Care for Cardiovascular Disease in China-Acute Coronary Syndrome

Supplementary Figures

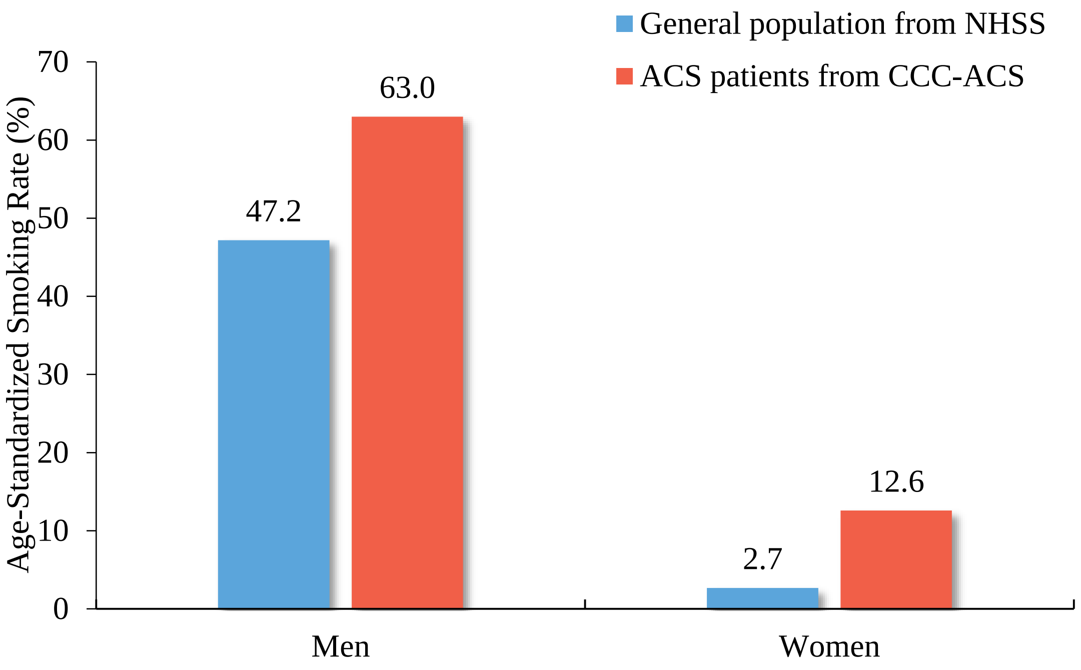

Supplementary Figure 1 Comparison of age-standardized smoking prevalence between ACS patients and the general population by sex

(Smoking prevalence was standardized using the age distribution of the general Chinese population obtained from the 2010 National Population Census [7])

Comparison of age-standardized smoking prevalence between acute coronary syndrome patients from CCC-ACS project and the general population from NHSS [8]. ACS: acute coronary syndrome; CCC-ACS: The Improving Care for Cardiovascular Disease in China-Acute Coronary Syndrome; NHSS: National Health Services Survey.

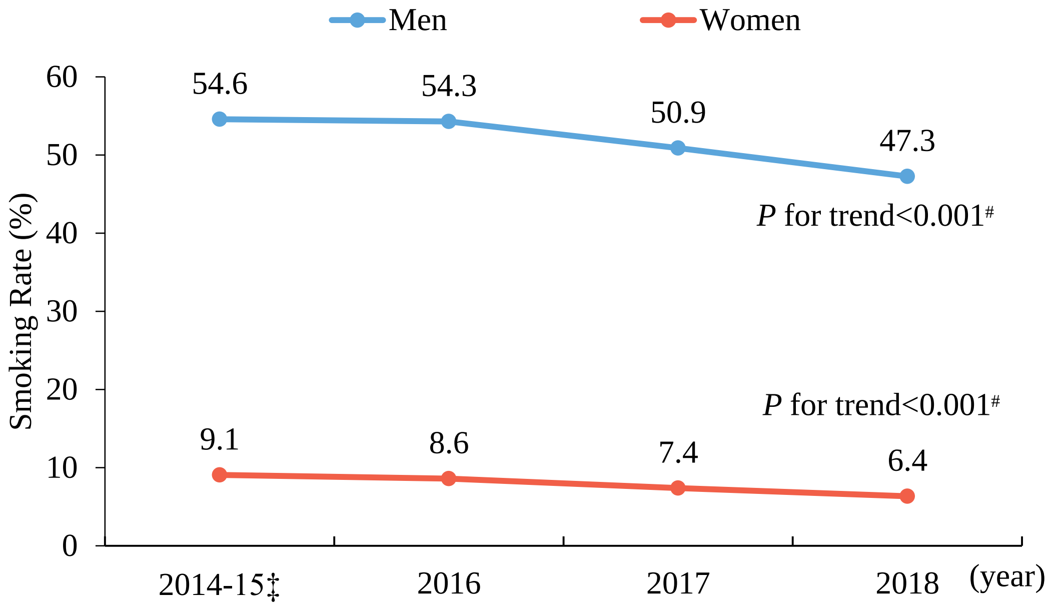

Supplementary Figure 2 Trends in smoking prevalence among ACS patients

‡ Since the project started in November 2014 and the data sample collected in 2014 is relatively small, we have merged data from 2014 and 2015.

Trends in smoking prevalence displayed by year from 2014 to 2018 for male acute coronary syndrome patients (blue) and female acute coronary syndrome patients (red). ACS: acute coronary syndrome.

Cochran-Armitage trend test

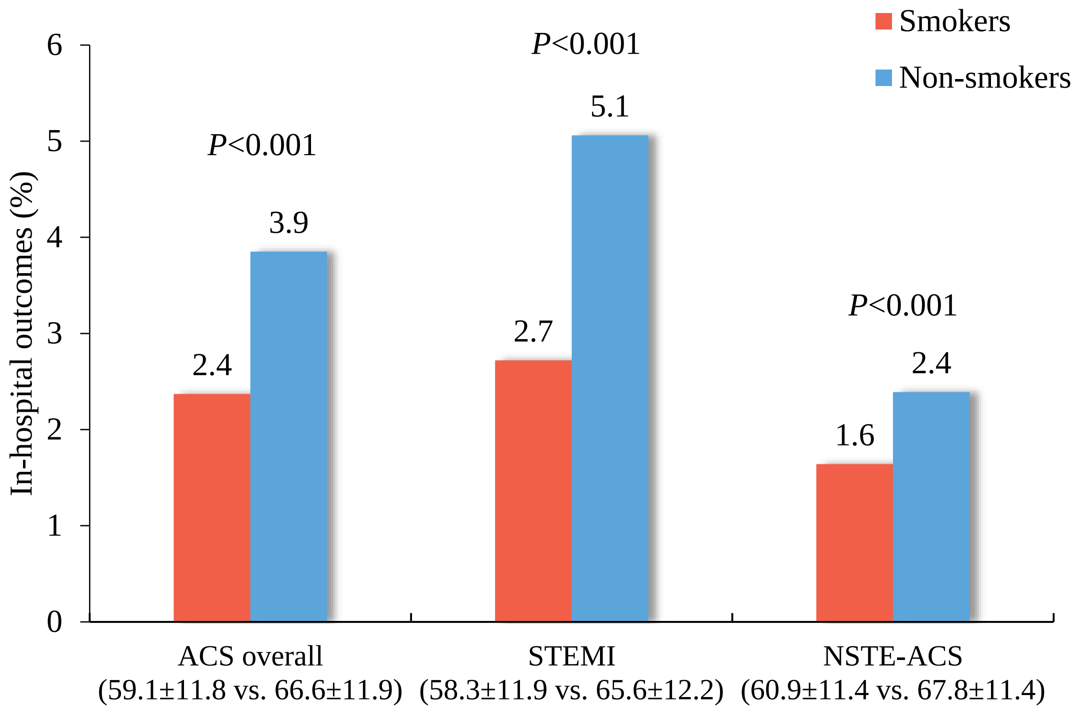

Supplementary Figure 3 In-hospital outcomes of smokers and non-smokers among patients with ACS (overall), STEMI, and NSTE-ACS

In-hospital outcomes according to smoking status among all patients with acute coronary syndrome, and among patients with specific subtypes of acute coronary syndrome. ACS: acute coronary syndrome; NSTE-ACS: non-ST-elevation acute coronary syndrome; STEMI: ST-elevation myocardial infarction

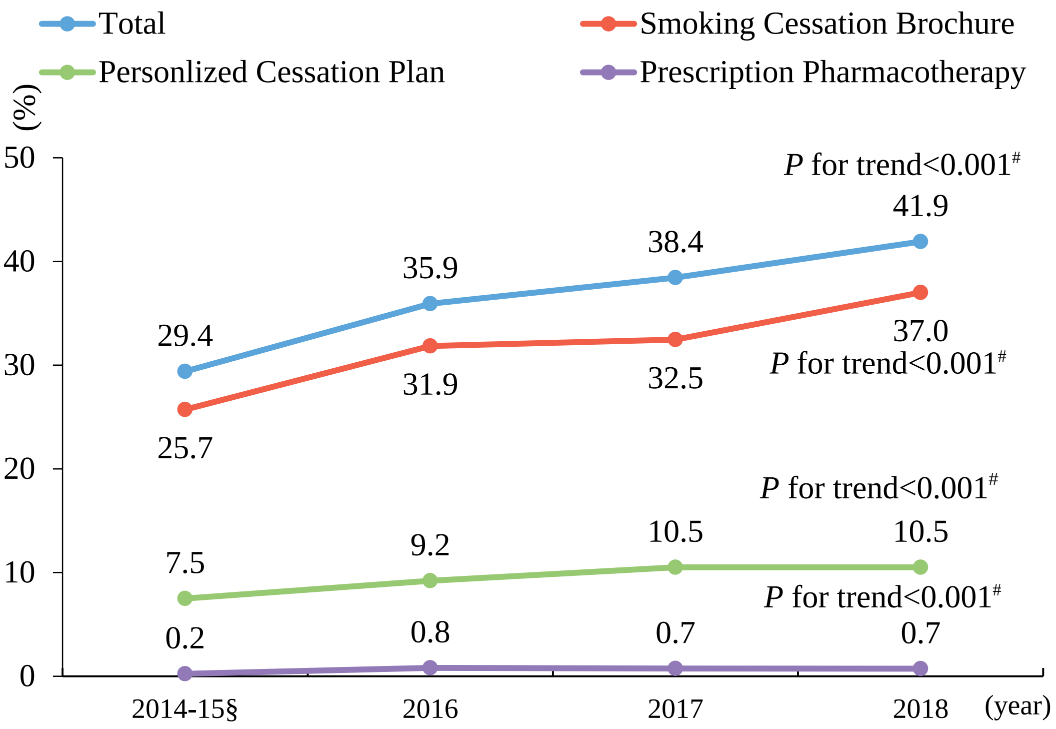

Supplementary Figure 4 Trends in proportions of patients receiving different smoking cessation interventions

§ Since the project started in November 2014 and the data sample collected in 2014 is relatively small, we have merged data from 2014 and 2015.

Trends in proportions of smoking patients receiving different smoking cessation interventions by year from 2014 to 2018.

Cochran-Armitage trend test

Supplementary References

1. **O'Gara PT, Kushner FG, Ascheim DD, et al.** 2013 ACCF/AHA guideline for the management of ST-elevation myocardial infarction: a report of the American College of Cardiology Foundation/American Heart Association Task Force on Practice Guidelines. *J Am Coll Cardiol*. 2013;61(4):e78-e140. DOI: http://dx.doi.org/10.1016/j.jacc.2012.11.019
2. **Amsterdam EA, Wenger NK, Brindis RG, et al.** 2014 AHA/ACC Guideline for the Management of Patients with Non-ST-Elevation Acute Coronary Syndromes: a report of the American College of Cardiology/American Heart Association Task Force on Practice Guidelines. *J Am Coll Cardiol*. 2014;64(24):e139-e228. DOI: http://dx.doi.org/10.1016/j.jacc.2014.09.017
3. **Ibanez B, James S, Agewall S, et al.** 2017 ESC Guidelines for the management of acute myocardial infarction in patients presenting with ST-segment elevation: The Task Force for the management of acute myocardial infarction in patients presenting with ST-segment elevation of the European Society of Cardiology (ESC). *Eur Heart J*. 2018;39(2):119-77. DOI: http://dx.doi.org/10.1093/eurheartj/ehx393
4. **Roffi M, Patrono C, Collet JP, et al.** 2015 ESC Guidelines for the management of acute coronary syndromes in patients presenting without persistent ST-segment elevation: Task Force for the Management of Acute Coronary Syndromes in Patients Presenting without Persistent ST-Segment Elevation of the European Society of Cardiology (ESC). *Eur Heart J*. 2016;37(3):267-315. DOI: http://dx.doi.org/10.1093/eurheartj/ehv320

5. **Levey AS, Stevens LA, Schmid CH, et al.** A new equation to estimate glomerular filtration rate. *Ann Intern Med*. 2009;150(9):604-12. DOI: http://dx.doi.org/10.7326/0003-4819-150-9-200905050-00006

6. **Chinese Society of Cardiology.** [Chinese guidelines for the diagnosis and treatment of heart failure 2014]. *Chin J Cardiol*. 2014;42(2):98-122. DOI: http://dx.doi.org/10.3760/cma.j.issn.0253–3758.2014.02.004

7. **National Bureau of Statistics.** Tabulation on the 2010 Population Census of the People's Republic of China. http://www.stats.gov.cn/tjsj/pcsj/rkpc/6rp/indexch.htm (accessed 10 January 2020).

8. **Wang M, Luo X, Xu S, et al.** Trends in smoking prevalence and implication for chronic diseases in China: serial national cross-sectional surveys from 2003 to 2013. *Lancet Respir Med*. 2019;7(1):35-45. DOI: http://dx.doi.org/10.1016/s2213-2600(18)30432-6
